# Retinal Vascular Changes during the Menstrual Cycle Detected with Optical Coherence Tomography Angiography

**DOI:** 10.1155/2021/5514575

**Published:** 2021-07-12

**Authors:** Linning Guo, Chenlei Zhu, Ziqi Wang, Zhiqiang Gao, Zongduan Zhang, Qintuo Pan

**Affiliations:** Eye Hospital and School of Ophthalmology and Optometry, Wenzhou Medical University, Wenzhou, Zhejiang, China

## Abstract

**Purpose:**

To evaluate the effects of the menstrual cycle on the retinal vascular status of healthy women by optical coherence tomography angiography (OCTA).

**Materials and Methods:**

Healthy women with regular natural menstrual cycles of 28 to 30 days were recruited for this prospective study. The women's retinal vascular status was measured by OCTA at 3 time points: the early follicular, ovulatory, and midluteal phases of the menstrual cycle. The main outcome measures were foveal avascular zone (FAZ) parameters, perfusion density (PD) percentage in the superficial retinal capillary plexus (SCP), and PD percentage in the deep retinal capillary plexus (DCP). The mean arterial pressure (MAP), spherical equivalent (SE), best-corrected visual acuity (BCVA), intraocular pressure (IOP), and axial (AL) were also measured in a same menstrual cycle.

**Results:**

In total, 62 right eyes of 62 women were included in the study. The mean age was 27.0 ± 1.73 (range, 24 to 31) years, and the mean menstrual cycle was 28.90 ± 0.84 (range, 28 to 30) days. The mean values of the DCP-PD parameters were significantly decreased in the nasal and inferior ETDRS subfields during the ovulatory phase. The mean DCP-PD in the nasal ETDRS subfield in the early follicular, ovulatory, and luteal phases was 54.11 ± 2.85, 56.39 ± 3.03, and 55.70 ± 3.27, respectively. The mean DCP-PD in the inferior ETDRS subfield in the early follicular, ovulatory, and midluteal phases was 52.90 ± 3.30, 54.86 ± 2.51, and 55.21 ± 2.64, respectively. No significant differences were found in MAP, SE, AL, IOP, FAZ area, or other quadrants of PD parameters, and no significant correlation was found between parameters by OCTA and age, MAP,SE, axial length, or IOP.

**Conclusions:**

The DCP-PD decreased in the nasal and inferior ETDRS subfields during the ovulatory phase in our study. This may indicate the need to consider the menstrual phase when interpreting DCP-PD parameters by OCTA in healthy women.

## 1. Introduction

Several studies have shown that sex steroid hormone receptors are located in most human ocular tissues, including the cornea, iris, ciliary body, lens, conjunctiva, retina, and lacrimal and meibomian glands [1–5]. Additionally, various endocrine alterations during the menstrual cycle are believed to play a vital role in the vascular physiology and related hemodynamic changes [6, 7]. Epidemiological studies have shown that sex hormones act differently according to sex and increase the incidence of various ocular diseases including cataract, dry eye, and neovascular age-related macular degeneration [8–11]. Although some researchers have discovered specific relationships between hormonal fluctuations and ocular tissue variables [6, 11–16], other researchers hold opposite opinions [17]. Changes in the corneal thickness, choroidal thickness, intraocular pressure (IOP), and other parameters during the menstrual cycle have been recently reported [12–16]. However, to the best of our knowledge, alterations in the retinal vascular status during the menstrual cycle have not been addressed.

Optical coherence tomography angiography (OCTA) is a recently developed noninvasive imaging technique that provides depth-resolved, high-resolution visualization of the retinal microvasculature and automated or semiautomated quantitative analysis without dye injection. It allows us to easily screen the superficial retinal capillary plexus (SCP) and the deep retinal capillary plexus (DCP) [18–21]. The present study was performed to investigate the retinal vascular changes during the menstrual cycle in healthy reproductive women by OCTA. We considered that if any retinal structures or perfusion parameters are shown to be affected by the menstrual cycle, such data should be clinically interpreted with regard to the specific menstrual phase.

## 2. Materials and Methods

This prospective study was conducted from May 1, 2018, through August 31, 2018, at the Wenzhou Medical University Affiliated Eye Hospital. The protocol was approved by the Ethics Board of the Wenzhou Medical University and was performed according to the tenets of the Declaration of Helsinki for research involving human subjects. Signed informed consent forms were returned to the researchers before examinations were performed.

Healthy reproductive women with regular natural menstrual cycles of 28 to 30 days were recruited for this study, and the data from the right eye were selected for analysis. The menstrual cycle history was self-reported by all women and checked for accuracy. The first day (early follicular phase) of menstruation was determined as the day on which bleeding occurred. Because the peak luteinizing hormone (LH) concentration precedes ovulation by 24 to 36 hours, the peak LH concentration in urine was measured when ovulation occurred. All women were supplied with a kit containing five tests (ClearPlan; Unipath Diagnostics, Waltham, MA, USA) that measure the amount of LH in the urine. The test was performed by the participants at home every day until the LH peak occurred. When the test revealed occurrence of the LH peak, the participants were asked to immediately contact the investigators. The next day was determined as the beginning of the ovulatory phase, and 3 to 7 days before the next menstrual bleeding was determined as the beginning of the luteal phase. All the examinations were made at the first day of the early follicular phase, the first day of the ovulatory phase and 3 to 7 days before the next menstrual bleeding.

The inclusion criteria were an age of 15 to 40 years, best-corrected visual acuity (BCVA) of 20/20 or better, refractive error of <6 diopters (D) spherical equivalent (SE), and a regular menstrual cycle. The exclusion criteria were any ocular disease that would prevent examination of the cornea or retina states; a history of laser application or any ocular surgery; a history of smoking or alcohol consumption; any systemic disease such as diabetes mellitus or hypertension; and a history of any medication use within the previous 3 months, including oral contraceptives, hormonal therapy, or systemic vasoactive drugs.

**Figure 1:**
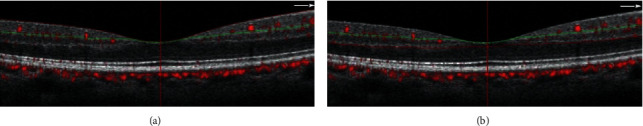
(a) The superficial retinal capillary plexus between the red and green lines and (b) deep retinal capillary plexus between the green and red lines were detected and separated automatically by using the OCTA instrument.

**Figure 2:**
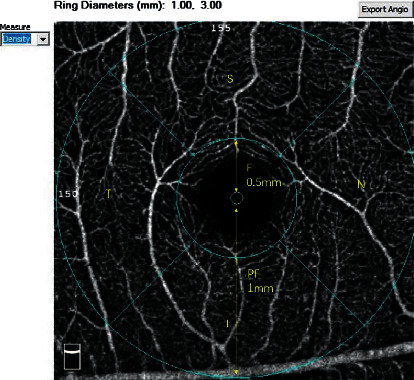
Automated superficial capillary plexus PD with overlay of ETDRS subfields. S: superior; I: inferior area; N: nasal; T: temporal. F: fovea; and PF : parafovea.

**Figure 3:**
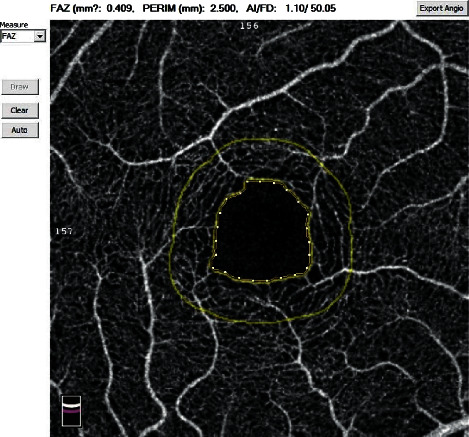
Automated FAZ parameters (area, perimeter, acircularity index, and fractal dimension).

The retinal vascular changes were imaged by using the RTVue XR Avanti OCT device with AngioVue software, version 2016.1.0.26 (Optovue, Inc., Fremont, CA, USA). A detailed ophthalmologic examination, including slit-lamp examination, fundoscopy, BCVA, SE, and intraocular IOP, was performed during each menstrual phase. IOP was measured using Goldmann applanation tonometry, and the axial length (AL) was measured by using a biometer (Lenstar LS 900; Haag-Streit AG, Köniz, Switzerland). All OCTA scans and measurements were performed by an experienced ophthalmic technician three times in the same session with a 10 min interval between each measurement. The intraobserver repeatability of the three measurements was analyzed, and the average value of three repeated measurements was used for the data analysis. A 3 × 3 mm cube scan centered on the fovea was acquired containing 304 × 304 A-scans. Each OCTA scan was automatically segmented to visualize the retinal perfusion density (PD) of the SCP and DCP. The SCP images were segmented with the inner boundary at the internal limiting membrane and the outer boundary at 10 mm above the inner plexiform layer (Figure 1(a)). The DCP images were segmented with the inner boundary 10 mm above the inner plexiform layer and the outer boundary 10 mm below the outer plexiform layer (Figure 1(b)). For more accurate measurement, the updated optical software (Optovue, Inc.) adopts three-dimensional projection artifact removal algorithms to remove “false” blood flow signals from the DCP. The five regions of interest were based on the Early Treatment Diabetic Retinopathy Study (ETDRS) contours and included the fovea (a circle with a 1 mm diameter) and the parafovea (an annulus with a 3 mm outer diameter and a 1 mm inner diameter centered at the fovea), which were divided into temporal, superior, nasal, and inferior quadrants (Figure 2). The automated software was also used to calculate the area (mm^2^) of the foveal avascular zone (FAZ) (Figure 3). All scans were reviewed to ensure correct segmentation and high image quality (quality index of >7). Poor-quality scans were excluded. Blood pressure measurements were obtained from the left arm using a sphygmomanometer. The mean arterial pressure (MAP) was obtained by calculating the diastolic pressure plus one-third of the pulse pressure. All examinations were performed within the same menstrual cycle.

## 3. Statistical Analysis

The data were analyzed using the SPSS statistical package, version 21.0 (SPSS Inc., Chicago, IL). For general statistical reporting, the mean values from each dataset were calculated with the standard deviations (SDs). A level of *p* < 0.05 was accepted as statistically significant. Visual acuity (VA) data were converted to the logMAR format for statistical calculations and analyses. The Kolmogorov–Smirnov test was used to check the normality of the sample distribution. The Kaiser–Meyer–Olkin measurement of sampling adequacy and Bartlett's test of sphericity were used to evaluate variance homogeneity. The data were analyzed using the repeated measures analysis of variance with post hoc pairwise comparisons corrected by the Bonferroni method. Greenhouse–Geisser correction and Huynh–Feldt correction were applied to adjust the degrees of freedom. Pearson's coefficient test was used to determine the correlation between parameters. The intraclass correlation coefficient (ICC), values ranging from 0 to 1, was calculated to determine the repeatability of consecutively measured OCTA. As the ICC value approaches 1, the repeatability of the measurement increases proportionally.

## 4. Results

Eighty-one volunteers participated in the study and 62 right eyes of 62 healthy women were finally enrolled and the other 19 volunteers did not finish the whole follow-up. None of the women reported any uncomfortable ocular or systemic symptoms. The Kaiser–Meyer–Olkin measure of sampling adequacy was greater than 0.50 (the actual values for each of the studied parameters ranged from 0.665 to 0.870). Bartlett's test of sphericity was <0.01 (the actual values for each of the studied parameters were <0.001). The mean age of the participants was 27.0 ± 1.73 (range, 24 to 31) years with a menstrual cycle of 28.90 ± 0.84 (range, 28 to 30) days. The BCVA in all eyes was 20/20 or above and remained constant during the study period. The mean parameters measured values of the 62 women at the 3 time points of the menstrual cycle are reported in Table 1. There was no significant difference in MAP, SE, AL, IOP, FAZ area, and all quadrants of SCP-PD parameters (Tables 1 and 2). The mean values of the DCP-PD parameters were significantly decreased in the nasal and inferior ETDRS subfields during the ovulatory phase. A significant difference was found in the nasal ETDRS subfield between the early follicular and ovulatory phases and between the ovulatory and luteal phases, but not between the early follicular and luteal phases (*p* < 0.001, *p* < 0.001, and *p* = 0.227, respectively). Similarly, a significant difference was reported in the inferior ETDRS subfield between the early follicular and ovulatory phase and between the ovulatory and luteal phase but not between the early follicular and luteal phase (*p* < 0.001, *p* < 0.001, and *p* = 0.106, respectively). Detailed data are presented in Table 3. No significant correlation between the OCTA parameters and age, MAP, SE, AL, or IOP was found. The statistical analysis demonstrated good intraobserver repeatability of three consecutive measurements in the current study (Table 4).

## 5. Discussion

Various and conflicting findings have been reported about the complex ocular alterations that occur during the menstrual cycle of women, ranging from the anterior segment parameters to choroidal changes, because the eye is also believed to respond to sex hormones. This has raised concern regarding various effects of female hormonal levels on the ocular circulation [1–5, 12–15]. Studies of cyclic ocular variations in women are scant and discordant. For example, Ulaş et al. [16] found that the choroidal thickness decreased significantly in the midluteal phase by using spectral-domain optical coherence tomography. Because estrogen receptors have been observed mainly in the ganglion cell layer of the retina and occasionally in the choroid [2], we hypothesized that more significant differences would be found in a retina-related study. However, little information about the retinal vascular changes that occur during the menstrual cycle is available. In this study, we used nonmydriatic, noninvasive OCTA to obtain retinal vascular measurements of women at three points in the menstrual cycle.

Several studies have shown that retinal microvascular changes can be affected by the AL, refractive errors, hypertension, and other factors [21–23]. Mo et al. [21] found that macular flow densities were negatively related to the AL but positively related to BCVA. Von Hanno et al. [22] reported that a higher MAP would reduce the central retinal artery caliber and elevate the central retinal vein caliber. Lee et al. [23] claimed that high blood pressure could affect the retinal microcirculatory structure and function, which may finally affect the retinal thickness. Although, there have been conflicting reports about changes in the blood pressure, associated with changes in the serum osmolality or not, during the menstrual cycle; for example, Chapman et al. [24] reported a decrease in MAP and the serum osmolality for vasodilation during the midluteal phase, while Vokes et al. [25] reported a decrease in the serum osmolality from the follicular phase to the luteal phase without any changes in the blood pressure changes. A hypothesis based on their findings was these changes might be associated with an increase in renal plasma flow and filtration, decreased systemic vascular resistance, or the corpora lutea which could release agents' impact on the hypothalamus to produce a reset osmotic threshold for vasopressin release [24, 25]. Moreover, Deschenes et al. [26] also reported that estrogen or estrogen and progesterone may help to increase retinal blood flow. Thus, we hypothesized that the AL or SE and the different phases of the menstrual cycle with alterations in hormone levels may induce retinal microvascular changes. In the present study, however, the MAP, BCVA, SE, IOP, AL, and SCP-PD did not significantly change during the menstrual cycle, and no significant correlation was found with age, MAP, SE, AL, or IOP in this homogenous group.

However, we did find that DCP-PD was significantly lower in the nasal and inferior ETDRS subfields during the ovulatory phase than in the early follicular phase and luteal phase. Conversely, we found no significant change in SCP-PD. The interrelationships of the retinal capillary plexuses are incompletely understood, and none of the currently available models could fully illustrate the true anatomical structure [27–32]. Some reporters have proposed the morphology of the SCP as a series of “hammocks” between a major artery and vein [28], while the morphology of the DCP appears totally different [29–31]. Garrity et al. [32] proposed that the DCP might be the primary site of venous outflow for the entire retinal microvasculature, while the SCP may not serve as distinct capillary units with an independent arterial supply or independent venous drainage. Thus, we believe the physiologic differences in structure and function between the SCP and DCP might cause them to be affected differently in the menstrual cycle. We only found differences in DCP-PD in the inferior and nasal subfields, not in the other quadrants, during the ovulatory phase. Chanwimol et al. [33] detected retinal vasculature changes in the third trimester of pregnancy by OCTA, and they found that DCP-PD was greater, especially, in the parafoveal, temporal, and inferior subfields. They speculated that this finding may represent gravity-dependent changes. But, whether this phenomenon can explain our results remains unclear because not only do hormones fluctuate in pregnancy but also much greater hemodynamic changes occur in response to the increasing circulatory demand to support the developing fetus over the course of about 9 months [33–36]; this situation seems totally different from that of our study participants. So, this issue remains to be studied in the future.

As mentioned earlier, Munaut et al. [2] reported that estrogen receptors are mainly located in the ganglion cell layer of the retina. Thus, we speculated that the hormone levels during different menstrual phases can explain the significant changes in DCP-PD that occur during the ovulatory phase with the absence of substantial differences in the early follicular and luteal phases. Studies have shown that estradiol is a potent vasodilator, and progesterone is assumed to have the opposite effect [36, 37]. Deschenes et al. [26] reported that estrogen or estrogen and progesterone together can increase retinal blood flow. Kızıltunç et al. [38] found a significant difference in PD of the whole macula of SCP and DCP in the pregnancy group when evaluating the macular and optic disc vessel density changes in pregnancy by OCTA. Also, the researchers proposed that hormonal changes might be one possible reason; for example, the increased levels of estrogen would lead to vasodilation and decreased vascular resistance, which may result in changes in ocular blood flow. As the estrogen concentration peaks before ovulation and the plasma progesterone concentration usually rise higher during the subsequent luteal phase [39, 40], some reporters voiced that the high estrogen levels increased the renin during the subsequent luteal phase [41, 42]. Thus, we proposed that the increased renin level would induce changes in vascular resistance to influence the blood pressure or ocular flow, which may be detected by OCTA. Measuring the concentrations of serum thyroid-stimulating hormone, luteinizing hormone, follicle-stimulating hormone, and prolactin may help to explore and confirm our speculation. However, because this was a noninvasive study, we could not obtain blood samples to search for relationships between hormone concentrations and retinal microvascular changes.

This study had some limitations. Our sample size was limited, and we did not measure the blood hormone concentrations and we could not analysis the peripapillary circulation or the retinal thickness. Further studies with larger sample sizes may help to determine the connections between ocular parameters and serum sex hormone concentrations. Further studies are also needed to investigate the effects of hormone concentrations, other vasoactive agents, and menstrual cycle irregularities. Despite these limitations, our study makes sense in the clinical context given the known menstrual cycle changes that occur in the retinal microvasculature.

In conclusion, this study demonstrated an apparent decreased DCP-PD in the nasal and inferior ETDRS subfields during the ovulatory phase. As the menstrual cycle spans a relatively long period of time in women and the hormones cyclically fluctuate, we tend to consider these findings as normal physiologic fluctuations without adverse effects on ocular function. Therefore, the menstrual phase must be taken into consideration if any significant changes are detected in the partial subfields of DCP by OCTA in women, especially in the nasal and inferior ETDRS subfields.

## Figures and Tables

**Table 1:** Main parameters measured during the menstrual cycle.

Parameter	Mean	SD	SE^b^	*p*
SE^a^ (diopter)				
EFP	−3.74	1.90	0.24	0.44
OVP	−3.75	1.91	0.24	
LP	−3.74	1.91	0.24	

AL (mm)				
EFP	24.98	1.00	0.13	0.16
OVP	24.98	0.99	0.13	
LP	24.96	1.00	0.13	

IOP (mmHg)				
EFP	15.16	1.74	0.22	0.15
OVP	14.93	1.47	0.19	
LP	15.38	1.19	0.15	

LogMAR VA				
EFP	0	0	0	—
OVP	0	0	0	
LP	0	0	0	

MAP (mmHg)				
EFP	87.54	4.79	0.61	0.32
OVP	87.76	4.86	0.62	
LP	87.88	4.64	0.59	

EFP: early follicular phase, OVP: ovulatory phase, LP: luteal phase, SE^a^: spherical equivalent, AL: axial length, IOP: intraocular pressure, VA: visual acuity, MAP: mean arterial pressure, SD: standard deviation, SE^b^: standard error.

**Table 2:** FAZ and SCP-PD OCTA parameters measured during the menstrual cycle.

Parameter	Mean	SD	SE^b^	*p*
FAZ area (mm2)				
EFP	0.32	0.12	0.02	0.34
OVP	0.33	0.12	0.01	
LP	0.32	0.12	0.01	

SCP-PD (%)				

Whole				
EFP	46.30	2.81	0.36	0.79
OVP	46.26	2.48	0.31	
LP	46.51	3.16	0.40	

Parafovea				
EFP	48.94	3.83	0.49	0.74
OVP	49.09	3.01	0.38	
LP	49.35	3.61	0.46	

Fovea				
EFP	16.04	5.63	0.71	0.30
OVP	16.53	5.60	0.71	
LP	16.58	5.59	0.71	

Temporal				
EFP	47.30	3.49	0.44	0.41
OVP	46.68	4.30	0.55	
LP	47.50	3.61	0.46	

Nasal				
EFP	47.79	4.59	0.58	0.67
OVP	48.37	3.43	0.44	
LP	48.14	4.79	0.61	

Superior				
EFP	50.65	4.14	0.53	0.65
OVP	50.89	2.86	0.36	
LP	51.13	3.73	0.47	

Inferior				
EFP	49.98	4.65	0.59	0.76
OVP	49.97	3.98	0.51	
LP	50.42	3.83	0.49	

SD: standard deviation, SE^b^: standard error, FAZ: foveal avascular zone, SCP: superficial retinal capillary plexus, PD: perfusion density, EFP: early follicular phase, OVP: ovulatory phase, LP: luteal phase.

**Table 3:** DCP-PD OCTA parameters measured during the menstrual cycle.

Parameter	Mean	SD	SE^b^	*p*
DCP-PD (%)				

Whole				
EFP	51.67	3.52	0.45	0.68
OVP	51.83	3.34	0.42	
LP	52.02	3.26	0.41	

Parafovea				
EFP	54.70	3.54	0.45	0.93
OVP	54.71	3.19	0.40	
LP	54.83	3.15	0.40	

Fovea				
EFP	31.43	8.37	1.06	0.70
OVP	31.74	8.50	1.08	
LP	31.69	8.14	1.03	

Temporal				
EFP	55.36	3.30	0.42	0.98
OVP	55.32	3.12	0.40	
LP	55.39	3.08	0.39	

Nasal				
EFP	56.39	3.03	0.38	<0.001
OVP	54.11	2.85	0.36	
LP	55.70	3.27	0.42	

Superior				
EFP	54.25	3.89	0.49	0.94
OVP	54.09	3.60	0.46	
LP	54.19	3.71	0.47	

Inferior				
EFP	54.86	2.51	0.32	<0.001
OVP	52.90	3.30	0.42	
LP	55.21	2.64	0.33	

SD: standard deviation, SE^b^: standard error, DCP: deep retinal capillary plexus, PD: perfusion density, EFP: early follicular phase, OVP: ovulatory phase, LP: luteal phase.

**Table 4:** Intraobserver repeatability for the parameters obtained by OCTA.

Parameter	ICC
FAZ area (mm2)	
EFP	0.982
OVP	0.938
LP	0.911

SCP-PD (%)	

Whole	
EFP	0.954
OVP	0.917
LP	0.901

Parafovea	
EFP	0.915
OVP	0.935
LP	0.917

Fovea	
EFP	0.971
OVP	0.877
LP	0.892

Temporal	
EFP	0.921
OVP	0.907
LP	0.884

Nasal	
EFP	0.925
OVP	0.947
LP	0.931

Superior	
EFP	0.882
OVP	0.903
LP	0.885

Inferior	
EFP	0.896
OVP	0.904
LP	0.923

DCP-PD (%)	

Whole	
EFP	0.887
OVP	0.913
LP	0.873

Parafovea	
EFP	0.825
OVP	0.900
LP	0.910

Fovea	
EFP	0.883
OVP	0.902
LP	0.885

Temporal	
EFP	0.934
OVP	0.907
LP	0.920

Nasal	
EFP	0.881
OVP	0.905
LP	0.917

Superior	
EFP	0.863
OVP	0.915
LP	0.924

Inferior	
EFP	0.927
OVP	0.921
LP	0.892

ICC, intraclass correlation coefficient; SCP: superficial retinal capillary plexus, PD: perfusion density, EFP: early follicular phase, OVP: ovulatory phase, LP: luteal phase, DCP: deep retinal capillary plexus.

## Data Availability

The data used to support the findings of this study are available from the corresponding author upon request.
